# Supplementary figures and images for: Sirtuin 1 alleviates alcoholic liver disease by inhibiting HMGB1 acetylation and translocation
Source: PeerJ. 2023 Nov 27;11:e16480. doi: 10.7717/peerj.16480 (PMC10688304; doi:10.7717/peerj.16480)

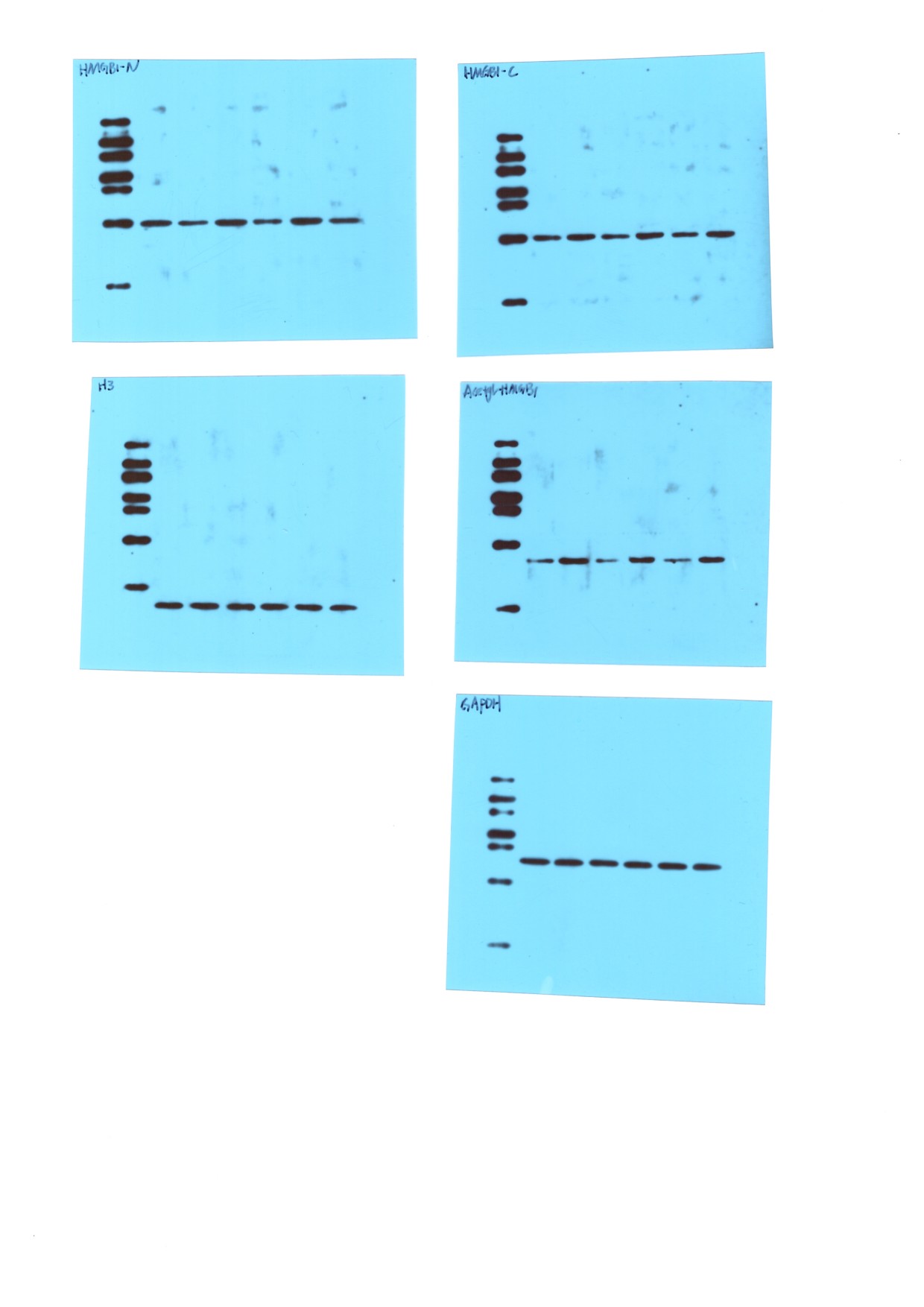

Supplement: Supplemental Information 1 [file peerj-11-16480-s001.zip › Figure 2G.jpg]

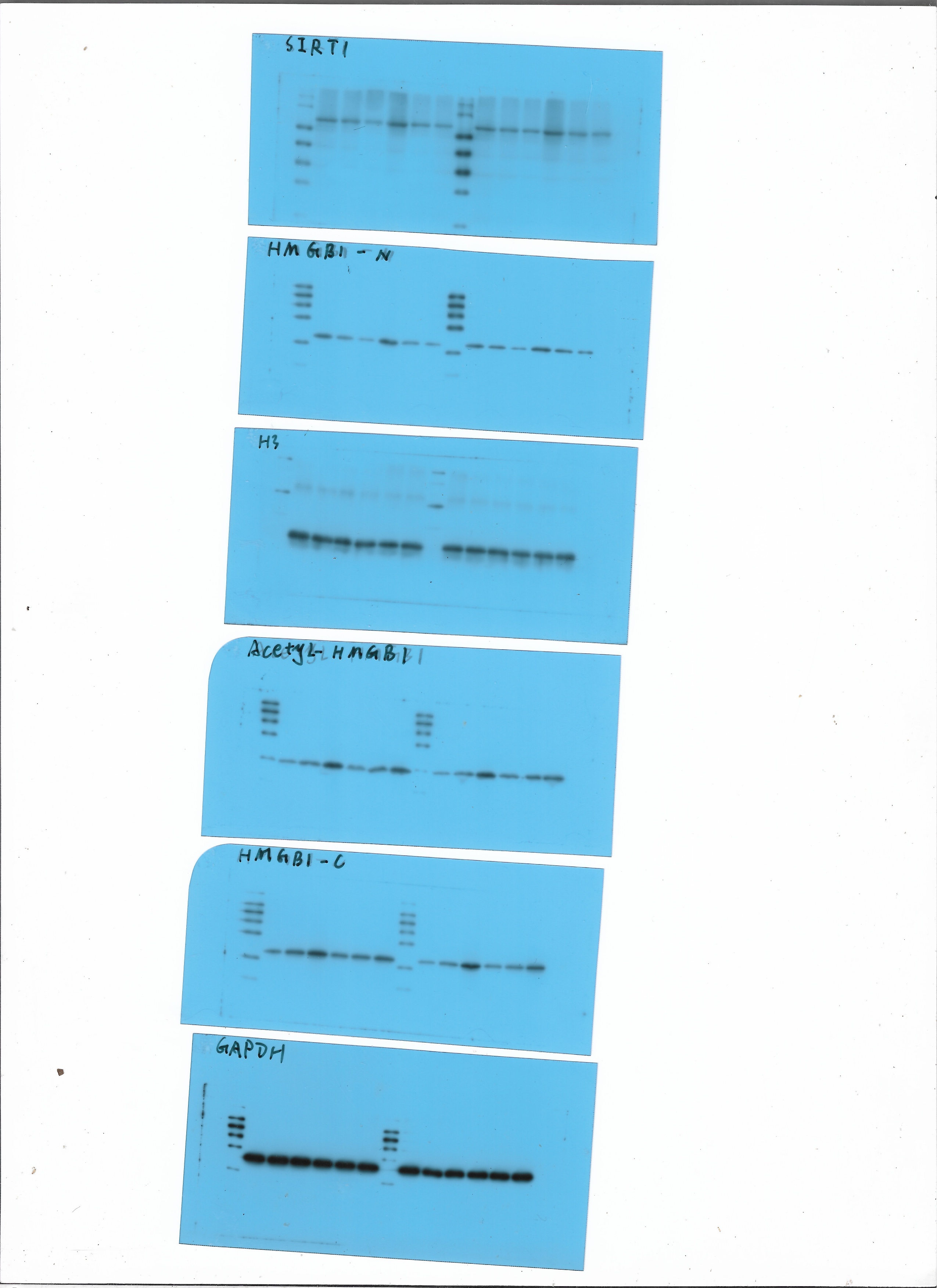

Supplement: Supplemental Information 1 [file peerj-11-16480-s001.zip › Figure 3A.jpg]

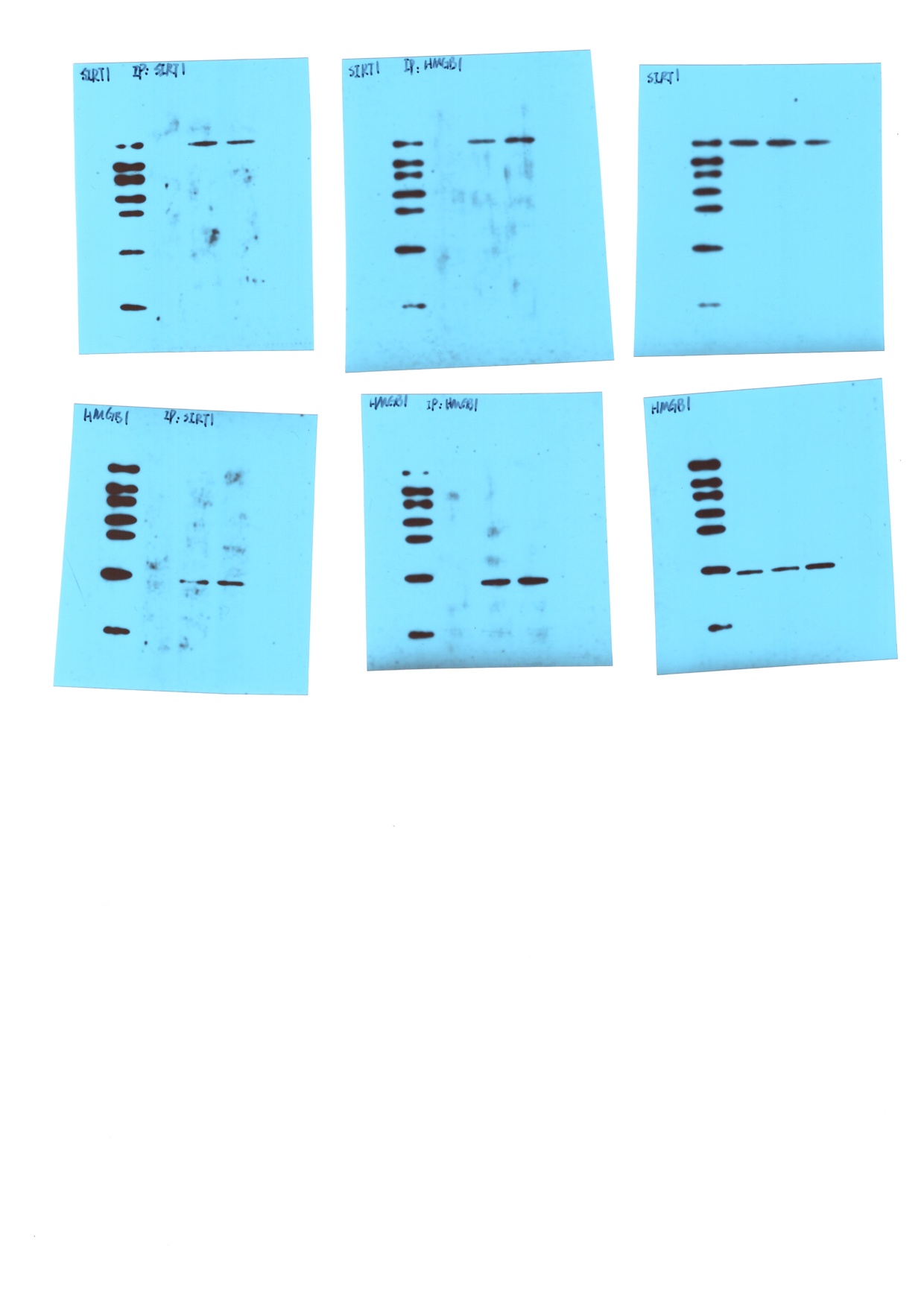

Supplement: Supplemental Information 1 [file peerj-11-16480-s001.zip › Figure 3B.jpg]

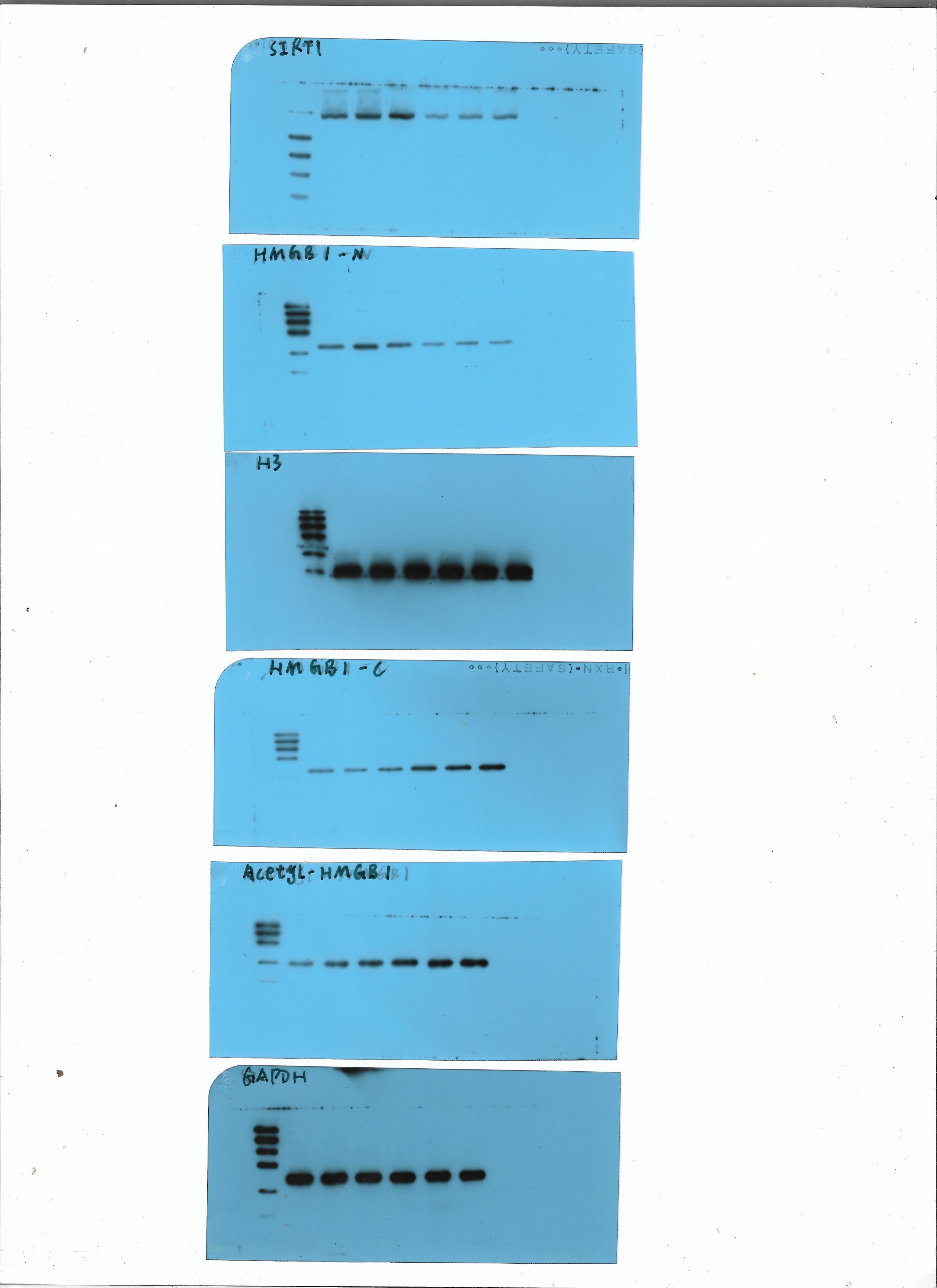

Supplement: Supplemental Information 1 [file peerj-11-16480-s001.zip › Figure 5J.jpg]

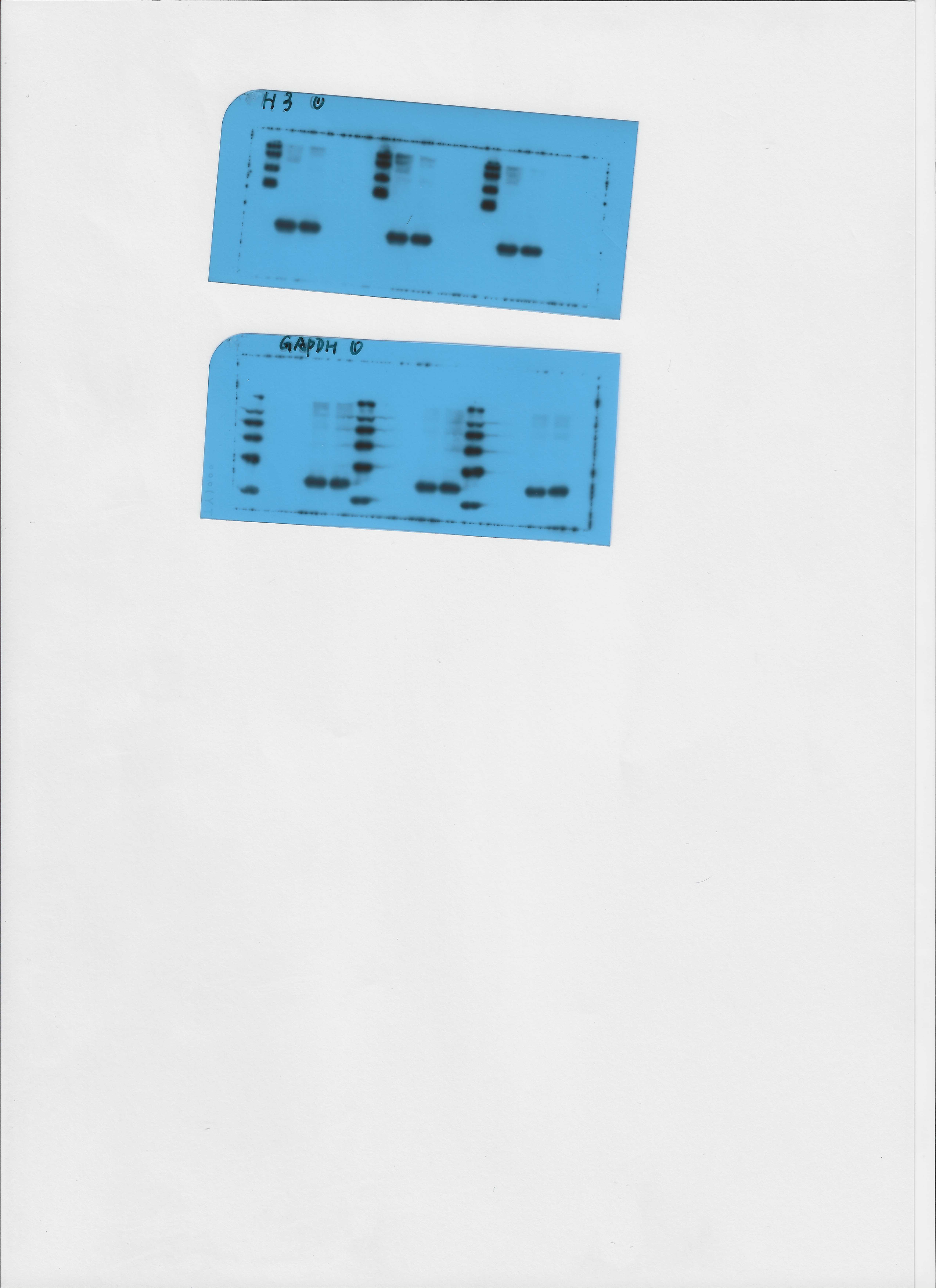

Supplement: Supplemental Information 2 [file peerj-11-16480-s002.zip › Supplementary figures/uncropped wb blots/Supplementary figure-1.tif]

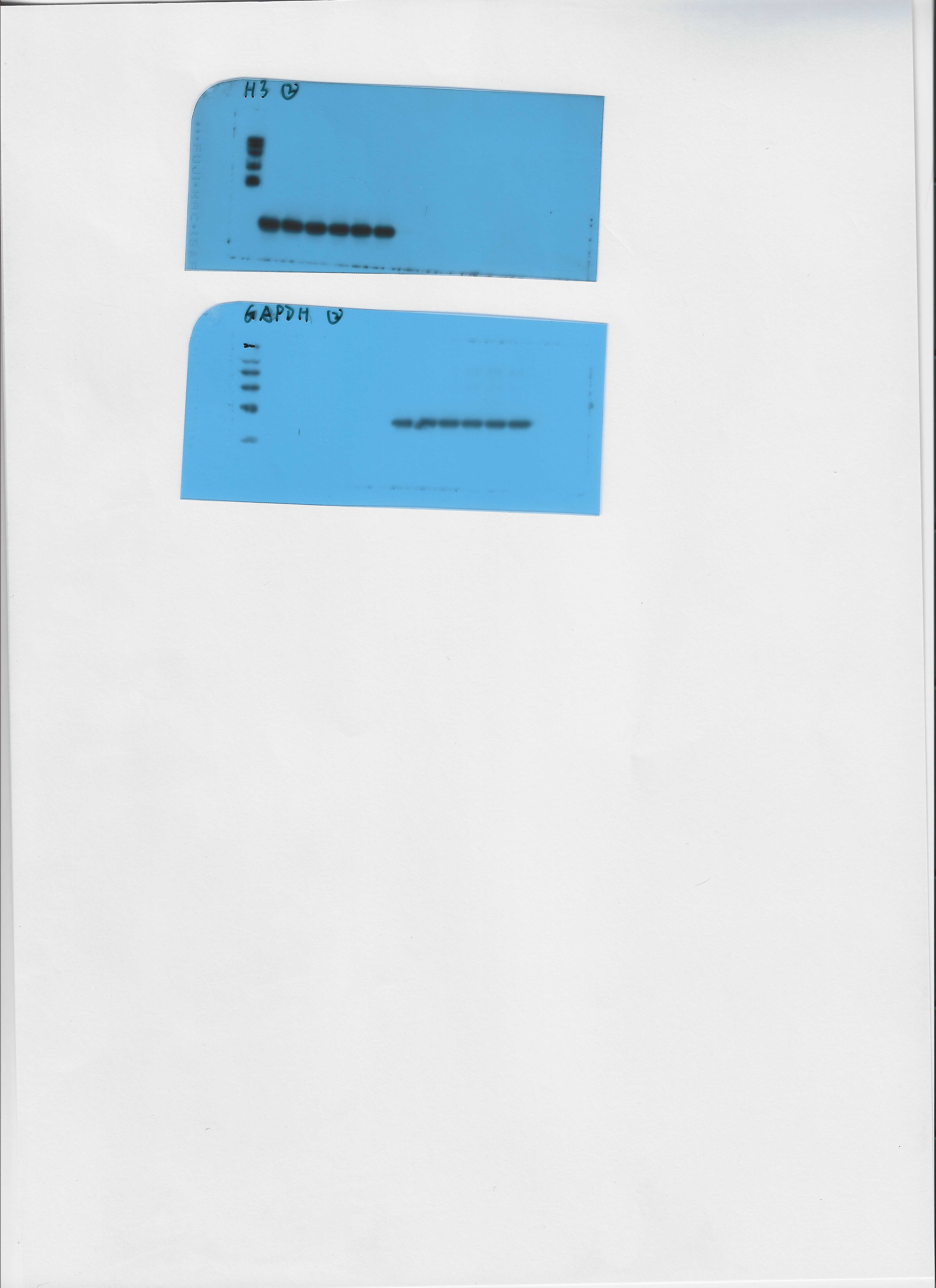

Supplement: Supplemental Information 2 [file peerj-11-16480-s002.zip › Supplementary figures/uncropped wb blots/Supplementary figure-2.tif]

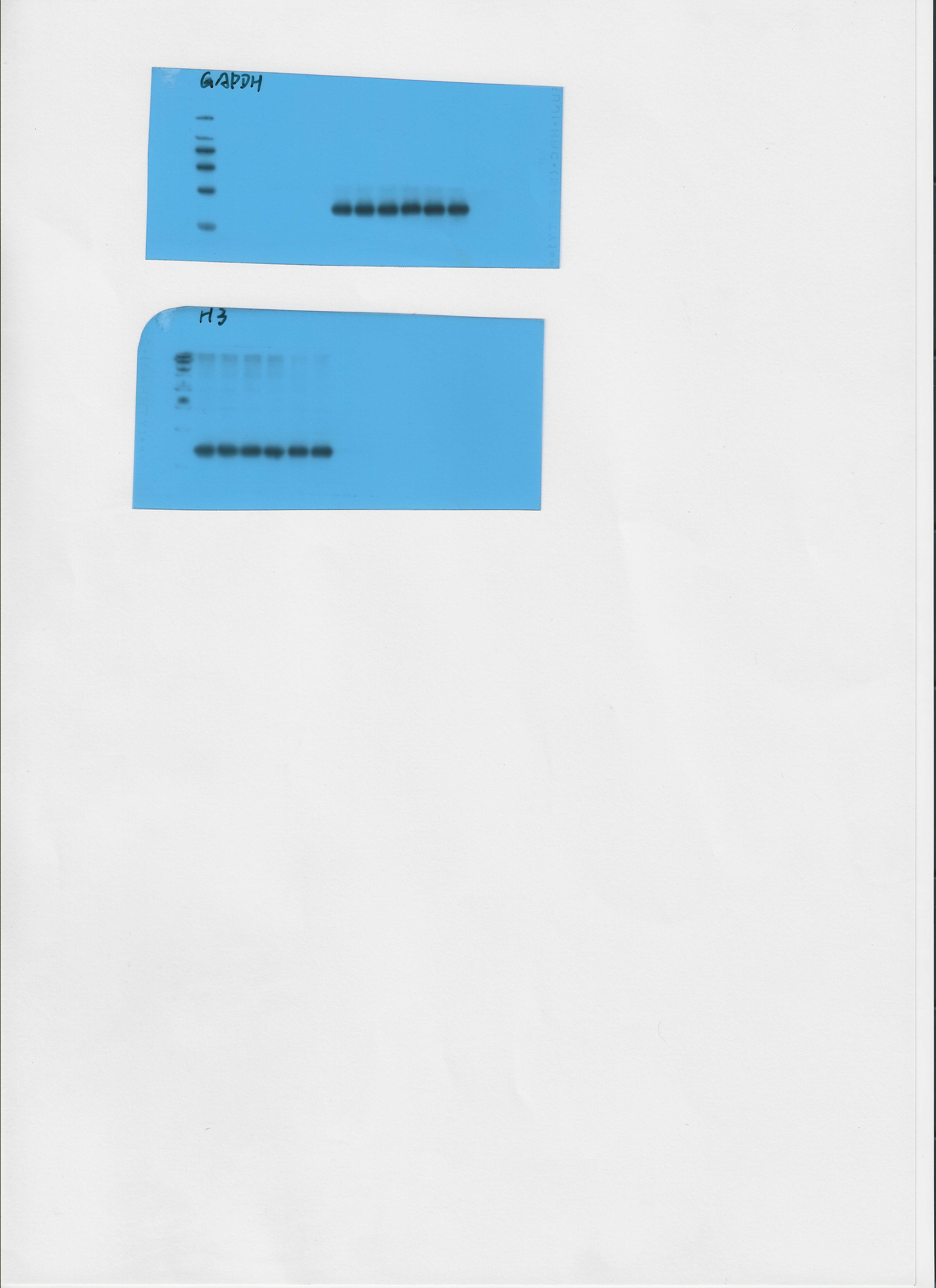

Supplement: Supplemental Information 2 [file peerj-11-16480-s002.zip › Supplementary figures/uncropped wb blots/Supplementary figure-3.tif]
