# Supplementary material for: Sirtuin 1 alleviates alcoholic liver disease by inhibiting HMGB1 acetylation and translocation
Source: PeerJ. 2023 Nov 27;11:e16480. doi: 10.7717/peerj.16480 (PMC10688304; doi:10.7717/peerj.16480)
Supplement: Supplemental Information 2 [file peerj-11-16480-s002.zip › Supplementary figures/Supplementary figures.docx]

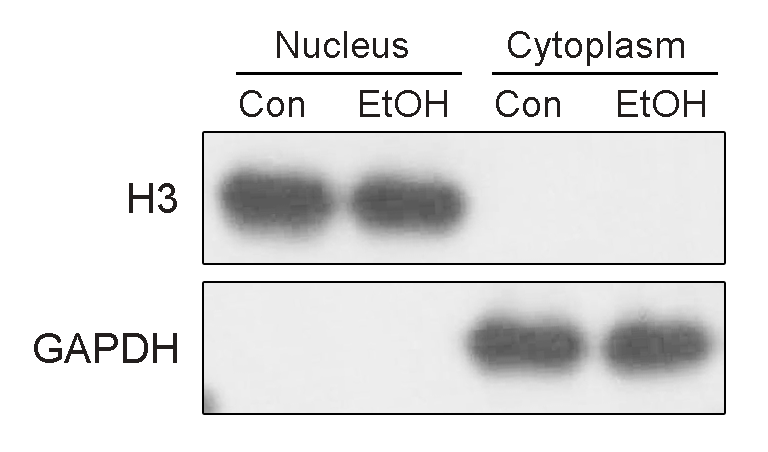


Supplementary Figure 1 Western blot analysis the expression levels of a nuclear marker, histone H3, and a cytosol marker, GAPDH.


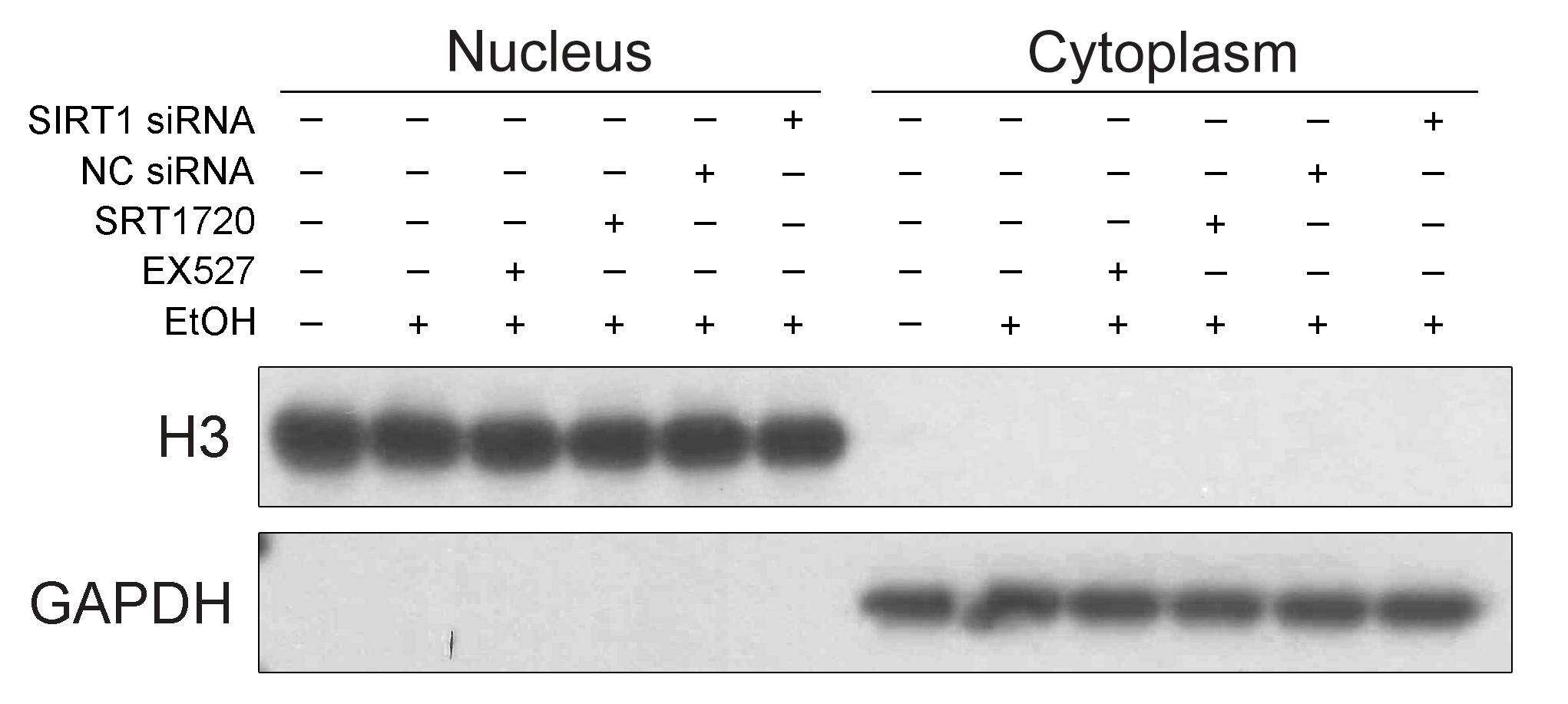


Supplementary Figure 2 Western blot analysis the expression levels of a nuclear marker, histone H3, and a cytosol marker, GAPDH.


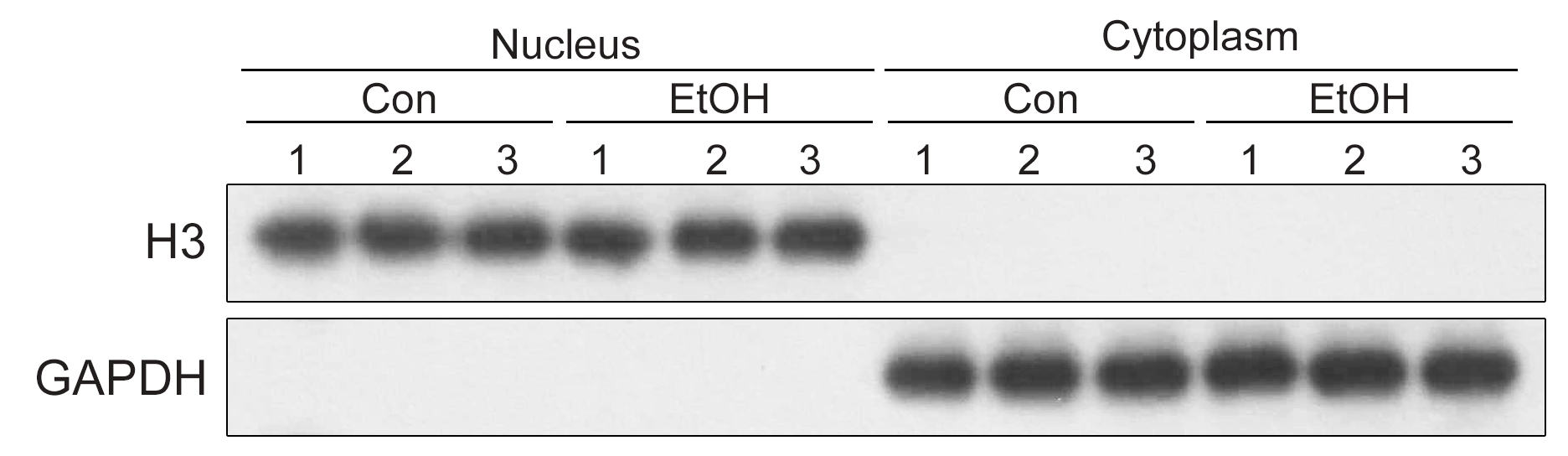


Supplementary Figure 3 Western blot analysis the expression levels of a nuclear marker, histone H3, and a cytosol marker, GAPDH.
